# Clinicopathologic Characteristics and Treatment Outcomes of Triple-Negative Breast Cancer at a Latin American Tertiary Hospital

**DOI:** 10.7759/cureus.91248

**Published:** 2025-08-29

**Authors:** Ricardo Metke, Lilian Torregrosa, Paola A Pinilla, Nathalie Tamayo, Laura C Lesmes

**Affiliations:** 1 Surgery, Hospital Universitario San Ignacio, Pontificia Universidad Javeriana, Bogota, COL; 2 Breast Surgery, Hospital Universitario San Ignacio, Pontificia Universidad Javeriana, Bogota, COL; 3 Oncology, Hospital Universitario San Ignacio, Pontificia Universidad Javeriana, Bogota, COL; 4 Epidemiology and Biostatistics, Hospital Universitario San Ignacio, Pontificia Universidad Javeriana, Bogota, COL; 5 Nursing, Hospital Universitario San Ignacio, Pontificia Universidad Javeriana, Bogota, COL

**Keywords:** colombia, complete pathological response, molecular subtyping, neoadjuvant chemotherapy, surgical management, triple negative breast cancer

## Abstract

Introduction

Triple-negative breast cancer (TNBC) lacks estrogen, progesterone, and HER2 receptors, represents a biologically aggressive subtype, and is frequently diagnosed at advanced stages. Regional data on its presentation and outcomes remain limited in Latin America. We evaluated the clinicopathologic features, treatment patterns, and survival of women with TNBC managed at a tertiary referral center.

Methods

A retrospective descriptive study was conducted of adult patients with pathologically confirmed TNBC who initiated and completed treatment at San Ignacio University Hospital, Bogotá, between August 2017 and June 2023. We reviewed medical records for demographic, tumor, and treatment variables, and we assessed patient responses to neoadjuvant chemotherapy (NAC) with the residual cancer burden (RCB) system. We used the Kaplan-Meier method to generate recurrence-free survival curves and compared survival in the subjects with and without pathological complete response.

Results

Of 1,083 breast cancer cases, 125 (11.5 %) were identified as TNBC. Most patients presented with stage II-III disease (104/125, 83.2 %) and node positivity (68/125, 54.4 %). NAC was administered to 90/125 women (72%); anthracycline-/taxane-based regimens predominated. Pathologic complete response after NAC occurred in 25/88 cases (28.4%). Forty-six patients had breast-conserving surgery (39.7%), whereas 70 patients (60.3%) underwent mastectomy. The estimated three-year recurrence/progression-free survival (PFS) was 79.4% [95% confidence interval (CI): 71.5%-88.2%]. The estimated three-year overall survival is 74.4% (95% CI: 66%-83.9%). Limited access to germline testing, advanced molecular profiling, and newer systemic agents was evident.

Conclusions

Within this Latin-American center, TNBC was infrequent yet predominantly diagnosed at advanced stages and carried substantial risks of relapse and death. Earlier detection, broader availability of genetic and molecular testing, and equitable access to emerging targeted and immune-based therapies are essential to improve outcomes in this high-risk population.

## Introduction

Breast cancer is the most common malignancy in women worldwide [1]. In Colombia, it represents 14.5% of all cancer diagnoses and causes 13.3 deaths per 100,000 inhabitants [1]. Global breast cancer mortality has declined due to advances in treatment and screening programs, yet survival rates vary significantly by histological and molecular subtype. 

Molecular classification stratifies tumors by receptor expression, guiding prognosis and therapy. Estrogen receptor (ER)-positive, progesterone receptor (PR)-positive, and human epidermal growth factor receptor 2 (HER2)-negative tumors account for approximately 70% of cases and typically respond well to endocrine therapy. HER2 overexpression occurs in approximately 20% of tumors, conferring an aggressive phenotype but amenable to targeted anti-HER2 agents. Triple-negative breast cancer (TNBC; defined by the absence of ER, PR, and HER2 protein expression) comprises 10% to 20% of cases globally and often presents at advanced stages [2-4].

TNBC is characterized by high histological grade and elevated mitotic index. Among patients receiving neoadjuvant chemotherapy (NAC), a complete pathological response (pCR) predicts improved long-term outcomes [2-4]. Triple-negative breast tumors have an overall recurrence rate of 3-17%, especially in the first years after treatment [2-4].

Given the aggressive nature and high mortality rate of TNBC, it is crucial to assess its prevalence, treatment patterns, and outcomes in our population to improve patient care. This study aims to describe the clinical characteristics and treatment outcomes of patients with TNBC at a tertiary breast cancer center in Latin America.

## Materials and methods

We conducted a retrospective descriptive study to characterize the clinical presentation, treatment, and outcomes of patients with TNBC from a cohort of 1,083 individuals diagnosed with breast cancer. Data were collected from patients treated between August 1, 2017, and June 30, 2023, at San Ignacio University Hospital in Bogotá, Colombia. Patient`s information was anonymized to protect their privacy. This study was approved by the Ethics and Research Committee of the Pontificia Universidad Javeriana and San Ignacio University Hospital (Approval No. FM-CIE-0204-20). 

The study population included all patients diagnosed with breast cancer and confirmed with a triple-negative molecular profile. Inclusion criteria were: (1) age ≥18 years; (2) initiation of treatment at San Ignacio University Hospital; (3) diagnosis of TNBC; and (4) completion of oncologic treatment at the institution. No exclusion criteria were applied, and we did not exclude any patients. 

Patient medical records were reviewed by the breast cancer functional unit. Extracted data included histological type, initial stage, molecular profile, date of diagnosis, date of last contact, treatments received, pathological response to NAC, and vital status at the time of data collection. Recurrence/progression was defined as any local, regional, or distant event after curative intent. All the patients with recurrence or progression had stage ≥IIA disease at diagnosis. Mortality was defined as the date of death recorded in the medical record, or, if unavailable, as the last date the patient appeared in the records of the General System of Social Security in Health, provided this date preceded the survival verification date. Patients lost to follow-up (n = 15) who were still registered in the General System of Social Security in Health on the survival verification date were classified as alive but were excluded from the recurrence/progression analysis. All information was entered into a standardized data collection format using the REDCap (Research Electronic Data Capture) system. We used statistical software R version 4.3.1 for the statistical analysis. We quantified the therapeutic response using the MD Anderson residual cancer burden (RCB) system [5]. 

Study data were collected and managed using REDCap electronic data capture tools hosted at San Ignacio University Hospital [6,7]. REDCap (Research Electronic Data Capture) is a secure, web- based application designed to support data capture for research studies, providing 1) an intuitive interface for validated data entry; 2) audit trails for tracking data manipulation and export procedures; 3) automated export procedures for seamless data downloads to common statistical packages; and 4) procedures for importing data from external sources.

A descriptive analysis was performed to assess disease characteristics and patient outcomes. Qualitative variables were reported as absolute frequencies and percentages. Quantitative variables were summarized using measures of central tendency and dispersion. Recurrence/progression-free survival (PFS) and survival rates were estimated with the 95% confidence interval (CI), and Kaplan-Meier survival curves were generated. We further described and compared recurrence/PFS and survival rates in the pCR and non-pathological complete response with a Log-Rank test.

## Results

Of the 1,083 patients diagnosed and treated for breast cancer between August 1, 2017, and June 30, 2023, at San Ignacio University Hospital, 125 (11.5%) were diagnosed with TNBC. We excluded patients with positive hormone receptor, positive HER2 receptors or patients without complete information regarding molecular profile. We included patients who had surgery, NAC, adjuvant chemotherapy, and radiotherapy at San Ignacio University Hospital. The median follow-up was 2.3 years (range: 26 days to 5.8 years). All patients were women (Table 1), and most (n=94, 75.2%) had no family history of breast cancer. The mean age at diagnosis was 59.6 years (standard deviation: 14.7 years).

**Table 1:** Patient characteristics

Variables		N	Percentage (%)
Gender	Female	125	100
	Male	0	0
Family breast cancer history	No history	94	75.2
	First degree family history	17	13.6
	second degree family history	13	10.4
	Unknown	1	0.8
Genetic testing	Yes	58	46.4
	No	67	54.6
Genetic study	Directed punctual mutations	6	10.3
	Genetic panel	52	89.7
Mutation	BRCA 1	8	13.8
	Others	11	19
	none	39	67.2
Histology type	Special invasive carcinoma - adenoid cystic features	2	1.6
	Special invasive carcinoma – apocrine features	4	3.2
	Special invasive carcinoma - other	4	3.2
	Special invasive carcinoma - metaplastic	10	8
	Non-special type - Ductal invasive carcinoma	105	84
Grade	I	6	4.8
	II	42	33.6
	III	74	59.2
	No information	3	2.4
Tumor diameter	T1	12	9.6
	T2	43	34.4
	T3	30	24
	T4	38	30.4
	Tx	2	1.6
Lymph node involvement	N0	55	44
	N1	49	39.2
	N2a	14	11.2
	N3	5	4
	Nx	2	1.6
Metastasis	M0	115	92
	M1	10	8
Stage	IA	11	8.8
	IIA	27	21.6
	IIB	25	20
	IIIA	20	16
	IIIB	28	22.4
	IIIC	4	3.2
	IV	10	8
KI67	0 - 20%	18	14.4
	21 - 50%	33	26.4
	60 - 100%	74	59.2

A genomic test was performed in 58 patients (46.4%), most receiving a complete genetic panel (52/58, 89.7%). In six/58 (10.3%) patients, we used a specific genomic test looking for a punctual BRCA mutation. Among all these, eight patients (13.8%) had BRCA1 mutations and 11 (19%) had other mutations. 

Histologically, 105 cases (84%) were classified as invasive carcinoma of no special type. Among the remaining cases, 10 (8%) were metaplastic carcinomas, four (3.2%) were apocrine carcinomas, two (1.6%) had carcinoma with adenoid cystic features, and one (0.8%) each of lobular carcinoma, pleomorphic lobular carcinoma, poorly differentiated canalicular carcinoma, and medullary carcinoma.

The majority of tumors were nuclear grade III (n=74; 59.2%), followed by nuclear grade II (n=42; 33.6%). Most patients presented with T2 tumors (n = 43; 34.4%), followed by T4 tumors (n = 38; 30.4%). Two patients were classified as Tx due to incomplete clinical data (1.6%). For lymph node involvement, 49 (39.2%) were classified as N1, and 55 (44%) were classified as N0; two cases were classified as Nx due to missing information (1.6%). At diagnosis, 115 patients (92%) had no evidence of distant metastasis. The proliferation index Ki-67 was greater than 60% in 74 cases (59.2%).

Regarding the clinical stage, 11 patients (8.8%) were diagnosed at stage I, while the majority (n = 104; 83.2%) were diagnosed at stage II or III. An additional 10 (8%) presented with stage IV disease at the time of diagnosis (Table 1).

Treatment characteristics

Table 2 summarizes treatment modalities. NAC was administered to 90 patients (72%), 26 (20.8%) underwent primary surgery, and nine (7.2%) received primary chemotherapy due to advanced disease without initial surgical indication. At diagnosis, 68 patients (54.4%) had lymph node involvement. Of the 90 patients who received NAC, only one did not proceed to surgery because the disease progressed during chemotherapy. Additionally, one patient who started on primary chemotherapy required an emergency mastectomy with axillary dissection.

**Table 2:** Treatment ACT: anthracycline, cyclophosphamide, and taxane; AC/TC: anthracycline, cyclophosphamide, taxane, and carboplatin; RCB: residual cancer burden.

Variables		N	Percentage (%)
Initial treatment	Primary surgery	26	20.8
	Neo adjuvant chemotherapy	90	72
	Primary chemotherapy	9	7.2
Breast surgery	Simple mastectomy	70	60.3
	Lumpectomy	46	39.7
Axillar surgery	Sentinel lymph node biopsy	39	33.6
	Axillary lymph node dissection	74	63.8
	Not done	3	2.6
Second surgery	Yes	10	8.6
	No	106	91.4
Neoadjuvant chemotherapy	ACT	46	51.1
	AC/TC	36	40
	Other	8	8.9
Pathological response	Complete pathological response	25	28.4
	RCB I	9	10
	RCB II	37	41.1
	RCB III	17	18.9
	Progression during neoadjuvant treatment	1	1.1
Adjuvant chemotherapy	Total	48	53.9
	Capecitabine	43	89.6
	Other	5	10.4
Radiotherapy	Yes	95	76
	No	30	24
Mortality during follow up	Alive	98	78.4
	Dead	27	21.6

Among those who underwent surgery (primary surgery, emergency surgery, and post-NAC surgery), 70 (60.3%) had a mastectomy, and 46 (39.7%) had a lumpectomy. Axillary procedures included axillary dissection in 74 patients (64.6%) and sentinel lymph node biopsy in 39 (33.6%). Initial axillary intervention was omitted in three cases: (1) a patient whose diagnostic lumpectomy confirmed metastatic disease and therefore did not return for a second operation; (2) a patient with substantial comorbidities who faced high surgical risk and declined axillary surgery; and (3) a patient who ultimately underwent axillary dissection during a re-intervention after the diagnostic lumpectomy.

Of patients who received NAC, 25/90 (27.8%) had T4 tumors. Chemotherapy regimens included anthracycline, cyclophosphamide, and taxane (ACT) in 46/90 cases (51.1%); anthracycline, cyclophosphamide, taxane, and carboplatin (AC/TC) in 36/90 (40%); and other regimens in eight cases (8.9%). One patient progressed to metastatic disease during NAC and was not taken to surgery.

Among NAC recipients who underwent surgery, 33/89 (37%) had lumpectomy, and 56/89 (62.9%) had mastectomy (with or without skin preservation). Sentinel lymph node biopsy was performed in 22/88 patients (25%), while 66/88 patients (75%) underwent axillary dissection. One patient experienced disease progression during NAC and therefore did not undergo either breast or axillary surgery. Another patient declined axillary surgery. Among all patients who had surgery (primary, emergency, or post-NAC), 10 of 116 (8.6%) required re-intervention because of positive sentinel lymph nodes or tumor-positive surgical margins.

Among the 26 (20.8%) of patients who had primary surgery, 13 (50%) underwent simple mastectomy, and 13 (50%) had lumpectomy. Sentinel lymph node biopsy was performed in 17 patients (65.4%) and axillary dissection in seven patients (26.9%) initially, based on lymph node involvement or intraoperative findings. Axillary surgery was initially omitted in two patients: one underwent a diagnostic lumpectomy and later required axillary dissection, placing them in the re-intervention group, while the other had confirmed metastases after the initial lumpectomy and did not proceed to axillary surgery. Overall, adjuvant radiotherapy was administered to 95 patients (76% of the cohort).

Pathological response and adjuvant treatment

The overall rate of pCR following NAC was 28.4% (25/88; we excluded one patient for whom RCB could not be calculated and one patient with progression during NAC). A significant residual disease burden (RCB class II or III) was observed in 61.4% of cases (54/88). RCB could not be calculated in one patient due to incomplete pathology data. pCR was achieved in 25 of 88 patients (28.4%) who received NAC. Fourteen of these patients (56%) had been treated with the ACT regimen and eight (32%) with AC/TC, while the remaining three received other chemotherapy regimens. Most patients who achieved pCR presented with stage IIB or more advanced disease (18/25); the other seven had stage IA or IIA disease at diagnosis.

Among those receiving NAC, 48/90 (53.3%) required adjuvant therapy. Of these, 43 (89.6%) received capecitabine, while five (10.4%) received other chemotherapy regimens.

Outcomes

**Figure 1:**
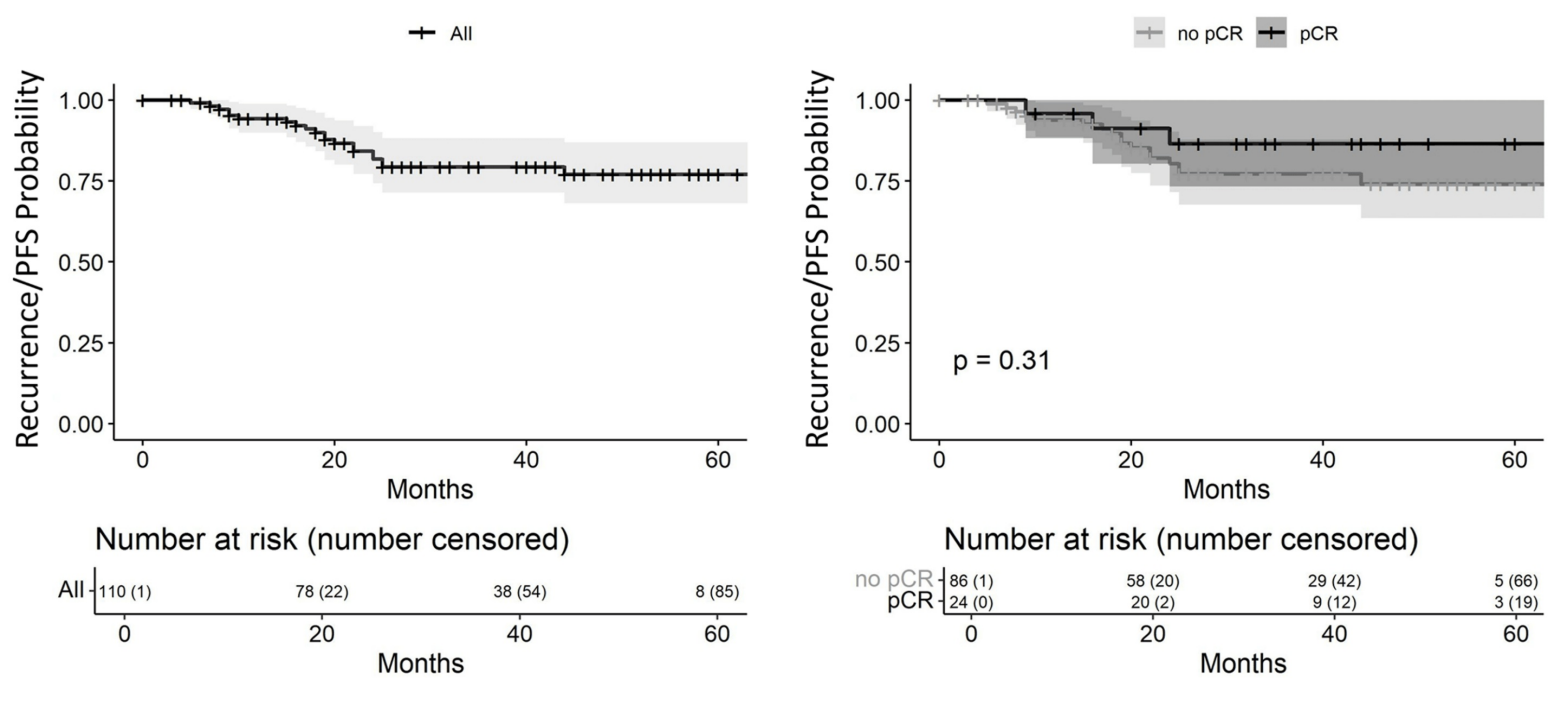
Kaplan-Meier recurrence/progression free survival curve in triple-negative breast cancer pCR: complete pathological response; PFS: progression-free survival.

Of 110 evaluable patients, 20/110 (18.2%) experienced disease recurrence or progression. Recurrence/PFS is shown in Figure 1. The estimated three-year recurrence/PFS for the entire cohort was 79.4% (95% CI: 71.5%-88.2%). Among patients who achieved pCR, the three-year recurrence/PFS was 86.5% (95% CI: 73.3%-100%), compared with 77.1% (95% CI: 67.7%-87.8%) in those without pCR (p = 0.31).

**Figure 2:**
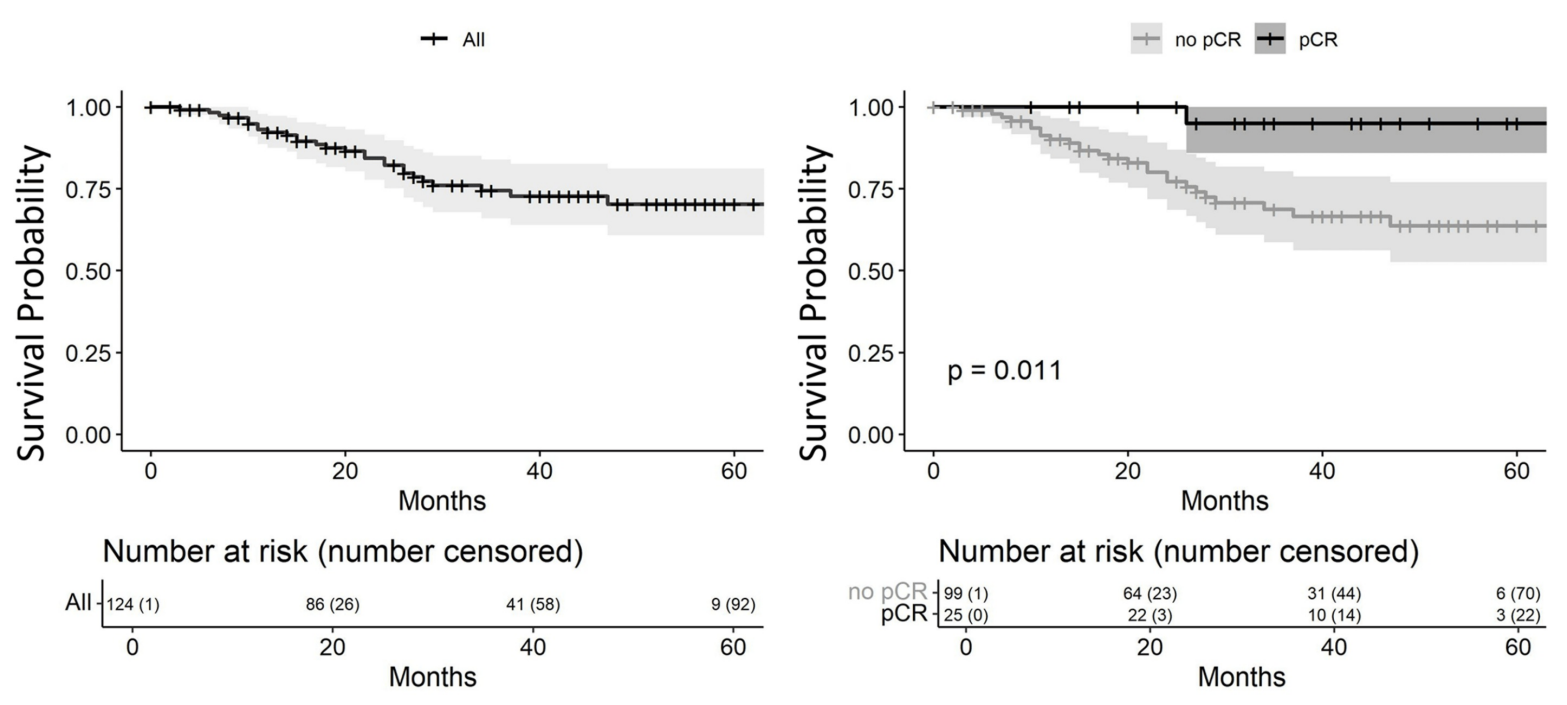
Kaplan-Meier survival curve in triple-negative breast cancer pCR: complete pathological response.

Overall survival (OS) by pCR status is presented in Figure 2. The estimated three-year OS for the full cohort was 74.4% (95% CI: 66.0%-83.9%). Patients with pCR had a three-year OS of 95.0% (95% CI: 85.9%-100%), whereas those without pCR had an OS of 68.6% (95% CI: 58.7%-80.3%), a statistically significant difference (p = 0.01).

## Discussion

The present study aimed to delineate the clinicopathologic characteristics and therapeutic outcomes of TNBC managed at a high-volume Latin American referral center. Three principal observations emerged. First, although TNBC constituted only a modest proportion of all breast cancers, most affected individuals presented with stage II-III disease and consequently commenced treatment with NAC. Second, a pCR was achieved in 28.4% of patients (a figure concordant with international series [8]) yet rates of recurrence/progression and mortality remained substantial. Third, outcomes appeared to vary alongside underlying tumor biology and potential socioeconomic and structural barriers, despite adherence to guideline-directed care.

Patient characteristics and tumor biology

TNBC accounts for 10% to 20% of breast cancers worldwide [2]. In our institutional cohort of 1,083 patients, 125 (11.5%) had TNBC, a prevalence consistent with the global literature. To ensure complete clinical and treatment data, we included only patients diagnosed and managed at San Ignacio University Hospital, a criterion that necessarily limited the cohort size.

TNBC is typically diagnosed in younger, pre-menopausal women and is associated with germ-line BRCA1 mutations in up to 75% of cases [9]. In the MD Anderson cohort, 57.1% of TNBC tumors harbored BRCA1 variants, and 23.3% harbored BRCA2 variants [8]. By contrast, the mean age at diagnosis in the present cohort was 59.6 years, and only half of the patients underwent genetic testing, primarily due to financial and administrative barriers. The older age distribution likely reflects local screening and referral patterns: screening programs at our institution primarily target older women, whereas younger women are seldom screened or referred promptly, leading to potential underdiagnosis in this subgroup. Among those tested, BRCA1 mutations were the sole alterations identified, suggesting a lower prevalence of hereditary predisposition, which may help explain the older age distribution observed, although a causal relationship cannot be confirmed in this study.

Molecularly, TNBC comprises at least seven subtypes delineated by gene-expression profiling (basal-like 1, basal-like 2, mesenchymal, mesenchymal stem-cell-like, immunomodulatory, luminal androgen-receptor-like, and unclassifiable) each exhibiting distinct therapy sensitivities [10]. Such profiling is not yet routinely available in most Latin American centers. Histologically, invasive ductal carcinoma predominated, consistent with global data; however, special subtypes, such as metaplastic carcinoma, were observed slightly more frequently, plausibly reflecting the institution’s referral status [2,11]. Tumor-infiltrating lymphocytes, a prognostic biomarker associated with superior outcomes, were not systematically assessed [12].

Systemic therapy and response evaluation

Systemic chemotherapy represents the cornerstone of TNBC management, with surgery constituting the second principal modality [13]. The systemic regimens used reflected contemporary standards of care during the study period. The ACT regimen was the most frequently prescribed, whereas AC/TC was selected for some patients at the treating oncologist’s discretion after consideration of individual clinical factors. An ACT regimen was administered to nearly three-quarters of patients, yielding a pCR rate of 28.4%, comparable to the 22% reported in the MD Anderson series [14]. The observed response rate is consistent with international experience and likely reflects the timely initiation of chemotherapy, close treatment monitoring, and prompt surgical intervention. A smaller subgroup received AC/TC and showed a slightly lower pCR rate, a finding that may be attributable to the limited sample size and the low prevalence of BRCA mutations in our cohort [15]. Most patients achieving pCR presented with stage IIB or more advanced disease, a pattern that probably reflects the scarcity of early-stage diagnoses in our setting.

Individuals achieving pCR or RCB I generally proceeded to breast-conserving surgery (BCS), whereas those with greater residual tumor burden underwent mastectomy followed by adjuvant capecitabine, conforming to contemporary guidelines [16-21]. During the study interval, poly (ADP-ribose) polymerase (PARP) inhibitors or immunotherapy were not approved for non-metastatic TNBC and, therefore, were not incorporated.

Local-regional treatment

Post-NAC surgical decisions were individualized but informed by chemosensitivity. Among NAC recipients, 36.6% ultimately underwent BCS; the remainder received mastectomy because of advanced T stage, extensive residual disease, or patient-selected risk-reducing surgery. Routine axillary dissection was performed in node-positive cases diagnosed before NAC, consistent with institutional policy during the study period. All BCS patients, and those undergoing mastectomy for tumors ≥ 5 cm or other high-risk features, received adjuvant radiotherapy, resulting in an overall irradiation rate of 76%.

Prognosis

TNBC commonly recurs or progresses within the first three years of follow-up, unlike other subtypes [14]. In our cohort, three-year PFS matched the 63% reported in external series [14]. These findings confirm timely treatment at our center and highlight the aggressiveness of TNBC. Although PFS was higher in patients who achieved pCR, the difference was not statistically significant, possibly owing to the small pCR subgroup.

OS in TNBC is likewise reduced during the first three years [14]. We observed a three-year OS of 74%, comparable to published data [14]. Despite this modest rate, the similarity to international figures suggests effective multidisciplinary care. Consistent with previous reports, patients with pCR showed a statistically significant OS advantage over those without pCR [14].

Stage at diagnosis and prognostic implications

Late-stage presentation paralleled global TNBC trends, wherein rapid tumor progression and younger age frequently situate patients outside standard screening algorithms [2,4]. Although Colombian screening guidelines encompass most women by age [4], restrictions in access and delayed clinical presentation continue to drive stage II-III diagnoses. Ten individuals presented with de novo metastatic disease and experienced the highest mortality, emphasizing the imperative of earlier detection.

Contextualization of outcomes

Our findings align closely with those reported by the National Cancer Institute of Colombia (2013-2016) [8], which documented a comparable mean age at diagnosis (57.3 years), proportion of locally advanced cases (61 %), NAC utilization (69.1 %), and pCR rate (22.6 %). Overall survival was likewise close in both series (71.5%vs 74%), highlighting both the intrinsically aggressive biology of TNBC and the possible negative consequences of delayed presentation, even within high-volume centers adhering to evidence-based protocols [8].

Limitations

Our study had several important limitations that constrain the interpretation and generalizability of its findings. Beyond the inherent drawbacks of a retrospective design and the loss of a small proportion of patients to follow-up, the study was conducted at a single high-volume referral center, which may attract biologically aggressive or surgically complex cases, thereby introducing referral bias. The sample size (125 patients accrued over nearly six years) limits statistical power, precludes robust subgroup analyses, and raises the possibility that modest effect sizes went undetected. Although descriptive survival estimates were provided, the absence of multivariable modeling prevents adjustment for confounding factors such as age, comorbidity burden, and treatment heterogeneity. Follow-up was relatively short (median, 2.3 years), which restricted the assessment of late recurrences and long-term toxicities. Genetic characterization was incomplete, as fewer than half of the cohort underwent germline testing, comprehensive next-generation sequencing was not performed, and tumor-infiltrating lymphocyte levels were not systematically recorded, all of which limit insight into the biologic drivers of outcome. Treatment patterns reflected the availability of drugs during the study window; newer agents, such as immune checkpoint inhibitors and PARP inhibitors for early-stage disease, were not accessible, potentially underestimating contemporary survival prospects. Lastly, quality-of-life metrics and functional outcomes were not captured, preventing a holistic appraisal of patient benefit beyond traditional oncologic endpoints. We could improve these limitations with a prospective clinical trial in which we could enhance data quality, minimize bias, and ensure standard treatment and follow-up. 

## Conclusions

This single-center analysis shows that, although TNBC represents a relatively small proportion of breast malignancies in our region, most patients present with stage II-III disease and experience appreciable rates of recurrence, progression, and mortality despite guideline-concordant treatment. Achieving a pCR in nearly one-third of cases treated with anthracycline- and taxane-based NAC underscores the potential for favorable outcomes, yet persistent disparities may be linked to delayed diagnosis, limited molecular testing, and restricted access to innovative therapies. To close these gaps, regional cancer-control strategies must prioritize timely access to screening and germ-line genetic testing to enable earlier detection and informed risk-reduction. It is also important to integrate routine molecular subtyping and emerging biomarkers (particularly tumor-infiltrating lymphocytes) into diagnostic pathways to guide personalized therapy. There should also be an equitable availability of novel systemic agents, including PARP inhibitors and immune checkpoint blockade, across both early-stage and metastatic settings.
